# The Role of Cyclophilins in Inflammatory Bowel Disease and Colorectal Cancer

**DOI:** 10.7150/ijbs.58671

**Published:** 2021-06-16

**Authors:** Lifang Liang, Rongxiao Lin, Ying Xie, Huaqing Lin, Fangyuan Shao, Wen Rui, Hongyuan Chen

**Affiliations:** 1 Department of Pathogenic Biology and Immunology, School of Life Sciences and Biopharmaceuticals, Guangdong Pharmaceutical University, Guangzhou 510006, Guangdong Province, PR China.; 2 Centrefor Novel Drug Research and Development, Guangdong Pharmaceutical University, Guangzhou 510006, Guangdong Province, PR China.; 3 GDPU-HKU Zhongshan Biomedical Innovation Plaform, Zhongshan 528437, Guangdong Province, PR China.; 4 Guangdong Engineering & Technology Research Center of Topical Precise Drug Delivery System, Guangdong Pharmaceutical University, Guangzhou 510006, Guangdong Province, PR China.; 5 Key Laboratory of Digital Quality Evaluation of Chinese Materia Medica of State Administration of TCM, Guangzhou 510006, Guangdong Province, PR China.; 6 Guangdong Cosmetics Engineering & Technology Research Center,Guangzhou 510006, Guangdong Province, PR China.; 7 Cancer Center, Faculty of Health Sciences, University of Macau, Macau SAR, China.

**Keywords:** inflammatory bowel disease, colorectal cancer, cyclophilins, CypA, CypB, CypD

## Abstract

Cyclophilins (Cyps) is a kind of ubiquitous protein family in organisms, which has biological functions such as promoting intracellular protein folding and participating in the pathological processes of inflammation and tumor. Inflammatory bowel disease (IBD) and colorectal cancer (CRC) are two common intestinal diseases, but the etiology and pathogenesis of these two diseases are still unclear. IBD and CRC are closely associated, IBD has always been considered as one of the main risks of CRC. However, the role of Cyps in these two related intestinal diseases is rarely studied and reported. In this review, the expression of CypA, CypB and CypD in IBD, especially ulcerative colitis (UC), and CRC, their relationship with the development of these two intestinal diseases, as well as the possible pathogenesis, were briefly summarized, so as to provide modest reference for clinical researches and treatments in future.

## Introduction

Cyps, a highly conserved protein family, is ubiquitous in prokaryotic and eukaryotic organisms. 1. This family generally possesses the activity of peptidyl prolyl cis-trans isomerase (PPIase), which be able to catalyze the cis-trans isomerization of proline residue peptide bonds. Indeed, due to the PPIase activity, Cyps has been demonstrated to play a role in protein folding 2, 3, and protein trafficking as well as chaperone activity 3, 4. Through this characteristic, on the one hand, Cyps exerts immunosuppressive effects. Cyps inhibits the activity of calcineurin through the interaction with cyclosporin A (CsA), and immunosuppressive drugs, via theirs PPIase active site, blocking the translocation of the nuclear factor of activated T cells (NF-AT) from the cytosol to the nucleus, and thus preventing the activation of T cells 5. On the other hand, Cyps inhibits cell proliferation and differentiation, promote apoptosis, etc. 6, 7. In addition, Cyps has also known to have relationship with the pathological processes of many diseases, such as viral infection 8, 9, cardiovascular diseases 10, inflammatory responses 11, 12 and cancers 13.

Up till now, there are at least 16 known human Cyps, which are structurally distinct 14, including Cyclophilin A (CypA), Cyclophilin B (CypB), Cyclophilin C (CypC), Cyclophilin D (CypD), Cyclophilin 40 (Cyp40), Cyclophilin NK (CypNK) etc. 15. Among them, most of the studies mainly focused on CypA, CypB and CypD. These members are found ubiquitously in different subcellular compartments. They have their own unique biological functions in cells consequently.

CypA, expressed in the cytosol, is the most abundantly expressed and first identified cyclophilin 16. Substantial evidence showed that intracellular CypA (iCypA) is secreted by several cell types, including vascular smooth muscle cells (VSMC), macrophages, and endothelial cells (EC), in response to inflammatory stimuli 17-24. Apart from the ordinary functions of Cyps, secreted CypA or named extracellular CypA (eCypA) participates in both inflammatory response and signal transduction 12, 23, 25. Additionally, the eCypA, through autocrine and paracrine, can mediate intercellular communications, serving as a chemokine that recruit inflammatory cells, as well as aggravate oxidative stress and inflammation 26, 27. Furthermore, studies suggested that high CypA expression correlates with poor outcome in patients with inflammatory diseases 20. Meanwhile, various reports have shown that CypA is upregulated in cancer 28-33 and is involved in diverse pathological processes of cancer development, such as synthesis of tumor-associated proteins, signal transmission of tumor cell growth, regulation of transcription factors, apoptosis, metastasis, and drug resistance 13, 16, 34-36. However, it is worth mentioning that a number of mechanistic details about CypA in IBD and CRC are still unknown and await further studies.

CypB is mainly located in the endoplasmic reticulum (ER), where it attenuates ER stress-induced cell injury by interacting with the ER-related chaperones 37. Cystolic CypB can also be stimulated by inflammation to form extracellular CypB (eCypB) 38-40. eCypB has multiple functions, including chemotaxis and signaling transduction 41-47. In addition, CypB is closely associated with the replication of hepatitis virus 48-52 and human immunodeficiency virus (HIV) 53, and found high expression in breast cancer, pancreatic cancer, glioblastoma, liver cancer and gastric cancer 54-59.

CypD, a component of the mitochondrial permeability transition pore (mPTP), is uniquely located in the mitochondrial matrix. It is responsible for regulating the opening of the mPTP 60. mPTP is a mitochondrial channel complex, primarily composed by several proteins, including voltage-dependent anion channel (VDAC), adenine nucleotide translocator-1 (ANT-1), and CypD 61, whose main function is to maintain the balance of mitochondrial respiratory chain 62. Under resting conditions, CypD shuts down the channel complex 62-64. When facing stimuli of hypoxia, calcium overload, and oxidative stress, CypD travels to the inner membrane and binds to ANT-1, which leads to mPTP sustained opening 63, 65-68, followed by mitochondrial membrane depolarization, mitochondria swelling, Ca^2+^ release, and eventually, cell death 62, 69-71. CypD is the basic component of mitochondrial function, and may contribute to regulating the opening state of mPTP to regulate inflammation 72 and cancer 73.

IBD has emerged as global diseases 74-80. New data suggest that the incidence and prevalence of IBD are affecting five million patients worldwide, and approximately 0.3% of the European and North American population suffer from IBD at the present time 79, 81, 82. IBD is a group of chronic, characterized by macrophages and neutrophils infiltration. Primarily, there are two clinical types of IBD: UC and Crohn's disease (CD) 83-88.

UC, the most common type of IBD, occurs mostly in the colon, affecting the entire intestinal tract in a discontinuous manner 89, 90. CD, on the other hand, mainly occurs in the rectum and affects part or all of the colon in a continuous manner 91, 92. According to statistics, in the countries with the highest incidence of IBD, the annual incidence of UC and CD was 24.3 and 12.7 per 100,000 person-years in Europe, 6.3 and 5 per 100,000 person-years in Asia and the Middle East, and 19.2 and 20.2 per 100,000 person-years in North America 81, 93, 94. The overall incidence is coalescing around a range between 15 and 5 per 100,000 person years for both UC and CD 94. It can be seen that as two of most common types of IBD diseases, the prevalence and incidence of UC and CD are rapidly increasing in the world. Although the researches on IBD have been growing and deepening in recent years, the exact etiology and pathogenesis remain unclear, which brings certain difficulties for clinical researches and disease treatments.

Clinical studies have shown that both UC and CD patients are at an increased risk for developing CRC compared with the general population 79, 95, 96. Furthermore, IBD can eventually develop into intestinal malignant tumor through intestinal adenoma by inducing oncogene instability, oncogene activation, and regulating cell proliferation. 97-100. CRC is a common malignant tumor of the digestive tract. Its incidence is increasing every year, with affecting approximately 1.23 million patients worldwide each year and accounting for almost 10% of all cancers 101-103. According to statistics, from 2015 to 2020, CRC became one of the leading causes of cancer deaths in China, ranking firmly in the top five cancer-related deaths 104. Its occurrence and development are affected by many factors, among which is closely related to inflammation and damage. Surgery is still the most effective treatment of CRC. Although great progress has been made in the prevention and treatment of tumors, its morbidity and mortality are still high 105. The main reason is that the disease has tumor features, such as invasion, metastasis, resistance and recurrence and other characteristics 106.

IBD and CRC are currently two of the common diseases in the intestinal tract, they are related but different. In the pathological development of two diseases, IBD can be regarded as one of the main causes of CRC, but they are different in disease characteristics, so this article will discuss the two diseases separately. In a number of studies, it has been found that CRC 107-109 and IBD 110-112 patients generally have high expression of Cyps. The previous research of our group shown that the lack of Cyclophilin J (CypJ) caused the loss of its protective effect in mouse colitis induced by dextransulfatesodium (DSS), and this is related to the ability of CypJ blocking the binding of ubiquitin chains, thereby negatively regulating nuclear factor kappa B (NF-κB) signaling 113. More relevant experiments are still needed to confirm the role of Cyps in enteritis and bowel cancer.

This article mainly review the expression of Cyps in IBD and CRC, as well as the possible mechanisms related to the occurrence and development of these two diseases, aiming to provide clues for finding an accurate and detectable biomarker for the diagnosis of the diseases.

## The relationship between Cyps and inflammatory bowel disease

### CypA

It was found that the expression of CypA was significantly increased in the crypt tissue 114, serum 110 and lymphocytes 111 of UC patients. Compared with the concentration of 2 ng/ml in the serum of health subjects, the CypA level in the serum of UC patients reached 6 ng/ml 110. Furthermore, CypA also showed characteristics related to UC disease progression. Expression of CypA in active UC patients was higher than that in remission UC patients 111. However, studies have further shown that CypA was not significantly elevated in colon tissue of UC patients, nor in serum of CD patients 110. This indicates that CypA plays an important role in IBD, especially in UC, but it is worth mentioning that the expression level of CypA may be different at different detection levels in UC patients. Simultaneously, in addition to the increase of CypA, the serum anti-CypA antibody in UC patients was also increased, and the expression level increased with the course of disease 111, 112, illustrating that the expression level of anti-CypA antibody may be positively correlated with the increase of CypA level, which suggests that anti-CypA also has a certain preoperative diagnostic value in inflammatory enteritis.

**Figure 1:**
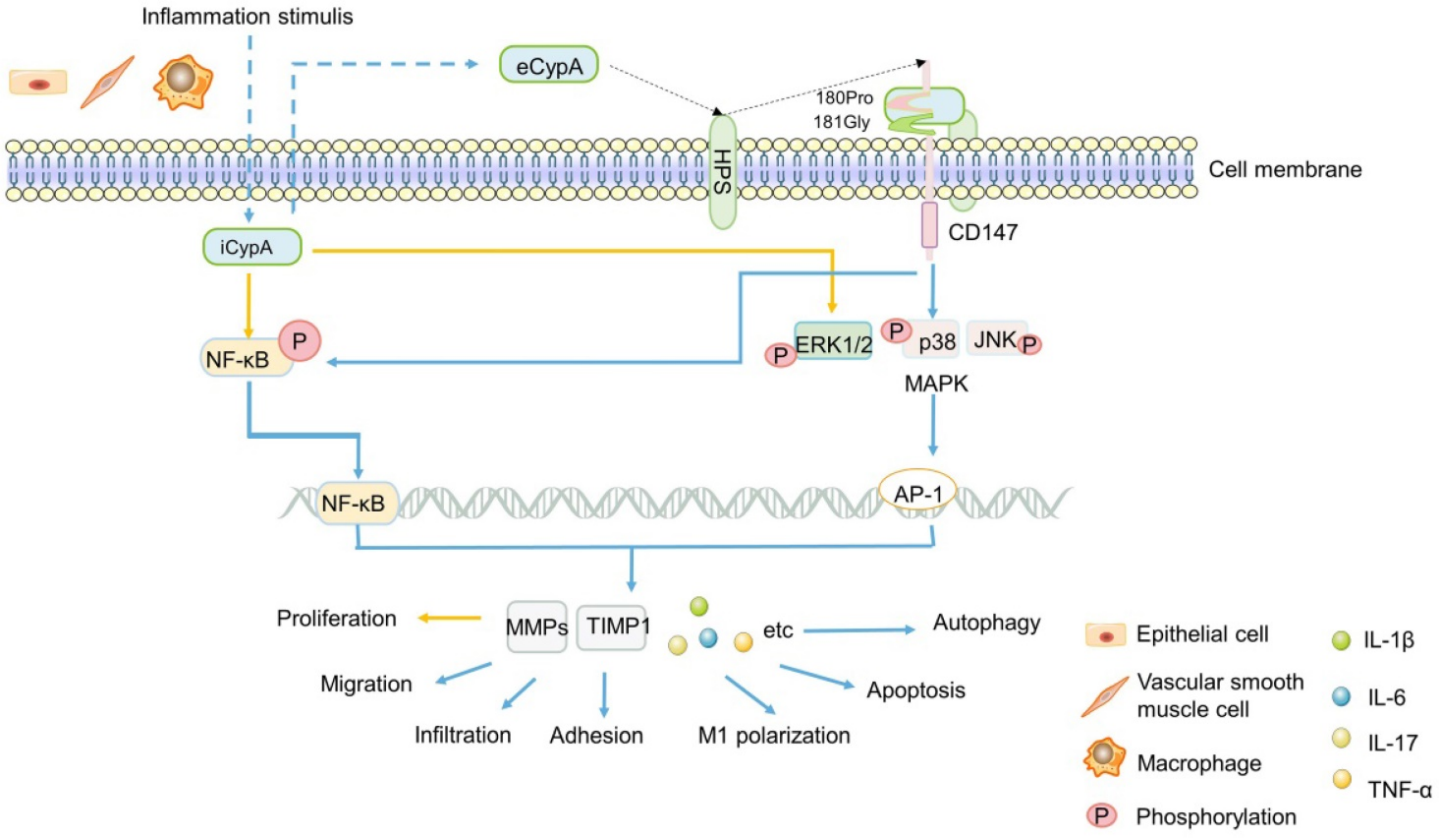
** A summary diagram of the possible mechanism of CypA in IBD.** Inflammation stimulates a variety of cells to secrete iCypA to form eCypA. The eCypA is presented to CD147 by HPS binding and entering into cells by CD147. Both eCypA and iCypA can regulate a variety of factors through NF-kB and MAPK pathways, and exert biological functions on immune cells regulating IBD.

Early studies found that eCypA was produced in macrophages stimulated by lipopolysaccharide (LPS) 18 (Figure 1), and it was found to be one of the stable reference genes for evaluating LPS-stimulated macrophages 14. Additionally, eCypA also upregulated and bound to macrophage surface differentiation cluster 147 (CD147) 115. In addition, eCypA induced the expression of inflammatory factors such as matrix metalloproteinase 9 (MMP-9), MMP-2, tissue inhibitor of MMP-1 (TIMP-1) 115, 116 or IL-1β, IL-6, IL-17 117, 118 through phosphorylation of (ERK1/2/JNK/P38) MAPK and NF-κB 117, 118, or induced autophagy 119, 120, apoptosis 120, M1 polarization 118, infiltration 12, chemotaxis and adhesion 121, 122 of monocytes/macrophages through these signals, which play a role in various inflammatory diseases (Figure 1). Others speculated that eCypA-induced autophagy in macrophages may be related to PI3K/Akt/mTOR signaling pathway, but no experimental study has been confirmed 95. However, it is noteworthy that some research results have proved that iCypA promoted the migration of dendritic cells 123 and the proliferation of macrophages 124 by inducing (ERK1/2) MAPK and NF-κB phosphorylation (Figure 1). This opposite effect of eCypA and iCypA on macrophages indicates that different forms of CypA may have opposite biological significance to the same cell by activating the same signal.

Not only macrophages, some researchers speculated that CypA is related to the obvious activation of lymphocytes in patients with UC, and the increase of CypA after lymphocytes activation may participate in the apoptosis of UC 111. Clinical studies showed that the levels of MMP-9 and TNF-α in UC patients were significantly increased with the increase of serum CypA, and the level of TIMP-1/MMP-9 complex in UC and CD patients were also significantly increased 110, suggesting serum CypA may influence MMPs and TIMPs in IBD patients. This result is consistent with the previous discoveries 115, 121, 122, speculating that the high expression of serum CypA in IBD may regulate the expression of TIMP-1/MMP-9 by activating ERK1/2, which promotes the pathogenesis and development of IBD, expecially UC (Figure 1). Further research is needed to confirm this hypothesis. In short, the difference between eCypA and iCypA lies in that the former may need to combine with the receptor CD147 and act on it first. Both receptor-mediated eCypA and iCypA, seems to activate MAPK, NF-κB and other signals to promote the proliferation or apoptosis, migration of a variety of immune cells and the expression of TIMP1, MMP9, MMP2, which may regulate IBD and other inflammatory diseases, but the specific mechanism is still unclear.

CD147, also known as extracellular matrix metalloproteinase inducer (EMMPRIN) or Basigin, is a transmembrane glycoprotein that can induce extracellular MMPs 125, 126. As matrix metalloproteinases, MMPs have been widely studied in the migration of inflammatory cells, cancer invasion and metastasis due to their universal function of degrading extracellular matrix components 121, 127, 128. In addition, CD147 is the cell surface receptor of eCypA and eCypB 121, 125, 129, 130. Heparans may be involved in the signal transduction induced by the binding of these two types of cyclophilins with CD147. It appears plausible that different heparan subtypes on the cell surface, namely sulfated glycosaminoglycans (GAG) and heparan sulfates (HPS, a subtype of GAG), which might facilitate eCypB-CD147 and eCypA-CD147 interaction by first binding eCypB and eCypA, respectively, then presenting them to CD147 (Figure 1) 131, 132. The interaction of eCypA-CD147/eCypB-CD147 and the transfer of eCypA/eCypB into the cells are promoted by the transduction activity of proline 180 (180Pro) and glycine 181 (181Gly) in the extracellular region of CD147, thereby activating the ERK signaling cascade 121, 125, 129, 130 (Figure 1).

Up till now, the importance of CD147 has been generally recognized by researchers 121, 125, 129, 130. In inflammatory, CD147 mediated the migration of monocytes/macrophages after binding to eCypA 121, 122 (Figure 1). In cancer, CD147 interacted with a variety of proteins, induces the secretion of MMPs, and promoted tumor invasion and metastasis 129, 133-136. Recent studies have shown that CD147 was significantly increased in intestinal mucosa of IBD patients and aggravated IBD inflammatory response by activating NF-κB 137. This indicates the important significance of CD147 in inflammatory diseases, and further confirms the results of previous studies 116-124 that eCypA firstly bound to CD147 on cell surface, and activated multiple signal pathways to regulate inflammatory cells, then promoted the expression of MMPs and other factors that can promote the occurrence and development of inflammation such as IBD, expecially UC.

Since the binding of CsA with CypA can inhibit its PPIase activity and exert immunosuppressive effect, it may have adverse effects on the normal immune function or disease treatment of the body 138-141. Therefore, a variety of CsA analogues binding to Cyps without causing immunosuppression have been developed clinically 142-146. In recent years, the researches on CypA have focused on the application of antibodies to diseases. Recombinant purified CypA proteins from different sources 117, 147 were used as immunogens to prepare polyclonal antibodies for the treatment of inflammatory diseases such as acute pneumonia 117 and sepsis 147. However, the application of anti-CypA antibodies in the treatment of IBD has not been found so far, which may indicate a new direction for the treatment of IBD and other inflammations in the future.

### CypD

**Figure 2:**
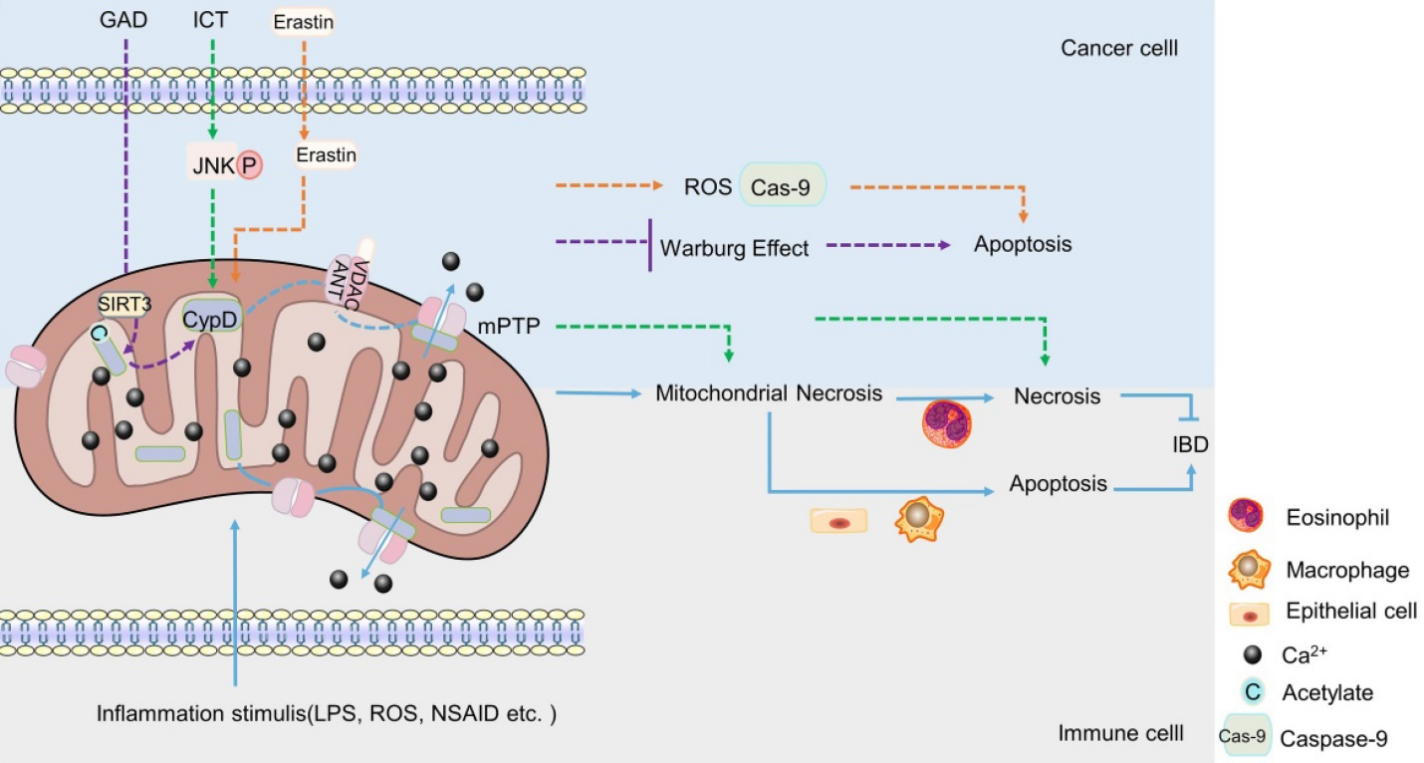
**The role of CypD in IBD and CRC.** When stimulated by inflammation, CypD travels to the inner membrane and binds to ANT-1, which leads to mPTP sustained opening, Ca^2+^ outflow, mitochondrial necrosis. The apoptosis of intestinal epithelial cells and macrophages can aggravate IBD, the necrosis of eosinophil can reduce IBD (Lower part). Various drugs or small molecules targeting mPTP can induce apoptosis or necrosis of CRC cells by inducing Warburg changes, necrosis and oxidative stress in mitochondria, respectively (Upper part).

The regulation of CypD on IBD is major related to mitochondrial permeability transition (mPT). *In vivo* and *in vitro* studies have shown that the inhibition or targeted deletion of CypD attenuated the mitochondrial necrosis of intestinal epithelial cells 148, macrophages 149 and eosinophils 150 induced by inflammatory stimuli such as non-steroidal anti-inflammatory drugs (NSAID), LPS and Ca^2+^ or oxidative stress, respectively, thereby regulating enteritis, which is related to the closure of mPTP after CypD deficiency (Figure 2). Interestingly, contrary to the results that CypD knockout or inhibition in macrophages and intestinal epithelial cells reduced inflammation, CypD knockout in eosinophils aggravated colon inflammation in mice. However, this may be related to the different regulatory roles of different target cells in IBD (Figure 2). In summary, the absence of CypD in intestinal epithelial cells, macrophages and eosinophils can protect cells from a series of mitochondrial reactions caused by the continuous opening of mPTP, such as mitochondrial membrane depolarization, increased reactive oxygen species and oxidative stress 66-68, thereby reducing cell death caused by mitochondrial necrosis (Figure 2). However, the decrease in the death of intestinal macrophages and endothelial cells plays a positive role in inflammation, while the decrease in the necrosis of eosinophils aggravates intestinal inflammation. Therefore, the difference in immune cells makes the lack of CypD also two sides for IBD.

### Other Cyps

In addition to CypA and CypD, other Cyps have also been found to play an important role in the development of IBD. CypJ, also known as PPIase-like 3 (PPIL3), is a newly discovered member of the cyclophilin family in recent years. It mainly exists in the cytoplasm and nucleus, and it also has PPIase activity 113. Previous studies have found that CypJ interacted with the Npl4 zinc finger (NZF) domain of TGF-β-activated kinase 1 binding protein 2/3 and the components of linear ubiquitin chain assembly complexes, blocking the ubiquitin chain binding, negatively regulating NF-κB signaling, thereby inhibiting DSS-induced colitis 113.

## The relationship between Cyps and colorectal cancer

### Cyps is high expression in CRC

In many studies, it has been confirmed that the expression of Cyps is significantly different between CRC and normal tissues. Over the years, many researchers have found that CypA 21, 32, 107, 151, CypB 108, 152, 153, CypE 154 and CypJ 155 in CRC tissue were increased expression compared with those in normal tissues or adjacent tissues. Even CypB in the serum of patients 107, 153 and CypA in different CRC cell lines *in vitro* 107, 153, were found to be overexpressed. Moreover, the high level of CypB has been confirmed to be related to the poor prognosis and low survival rate of patients 108, 152, 153. Some studies also found that the high expression of CypA in colon cancer was accompanied by the up-regulation of CD147 expression, and the expression changes of the two were consistent. It is suggest that CD147 may be positively correlated with the over-expressed of CypA in colon cancer tissues, which provides clues for further exploring the mechanism of CypA in CRC. In summary, compared with non-cancerous tissues, the expression of Cyps in CRC is generally up-regulated, suggesting that the expression of Cyps may be related to the occurrence of CRC.

Besides the markedly high expression of Cyps in CRC, the expression of Cyps is closely correlated with the progression of CRC. The expression of Cyps increased with the decrease of CRC differentiation, the occurrence of lymph node metastasis 21, 156, TNM (tumor, node, metastases) stage and tumor invasion 109, 155. Consistent with these experimental results, in addition to the direct study of CRC patients with pathological tissues, there were also through the establishment of early submucosal non-invasive and invasive CRC rat tumor model, proteomics analysis found that compared with non-invasive CRC and normal control group, invasive CRC CypA protein expression increased significantly 157. Interestingly, Yeonghwan Kim's team also studied the relationship between CypB expression and tumor progression, but did not find any relevance between CypB overexpression and the grade or development of colon cancer 152, which seems to be in contradiction with the previous researches that found the expression of CypB in CRC with lymph node metastasis was significantly increased 156. Yeonghwan Kim et al. 37 believed that it was related to the fact that CypB was mainly distributed in ER, and its expression might be mainly affected by ER oxidative stress rather than tumor invasion and metastasis. Urgently, the expression level of CypB in the progression of CRC needs more experiments to illustrate, but clearly, these results can be confirmed that the general high expression of Cyps may be closely related to the occurrence, development and metastasis of CRC.

## The possible mechanisms of Cyps in CRC

In addition to its high expression in CRC, different members of Cyps can participate in the development of CRC in different ways. The mechanism of Cyps in CRC will be briefly reviewed in the form of family members.

### CypA

**Figure 3:**
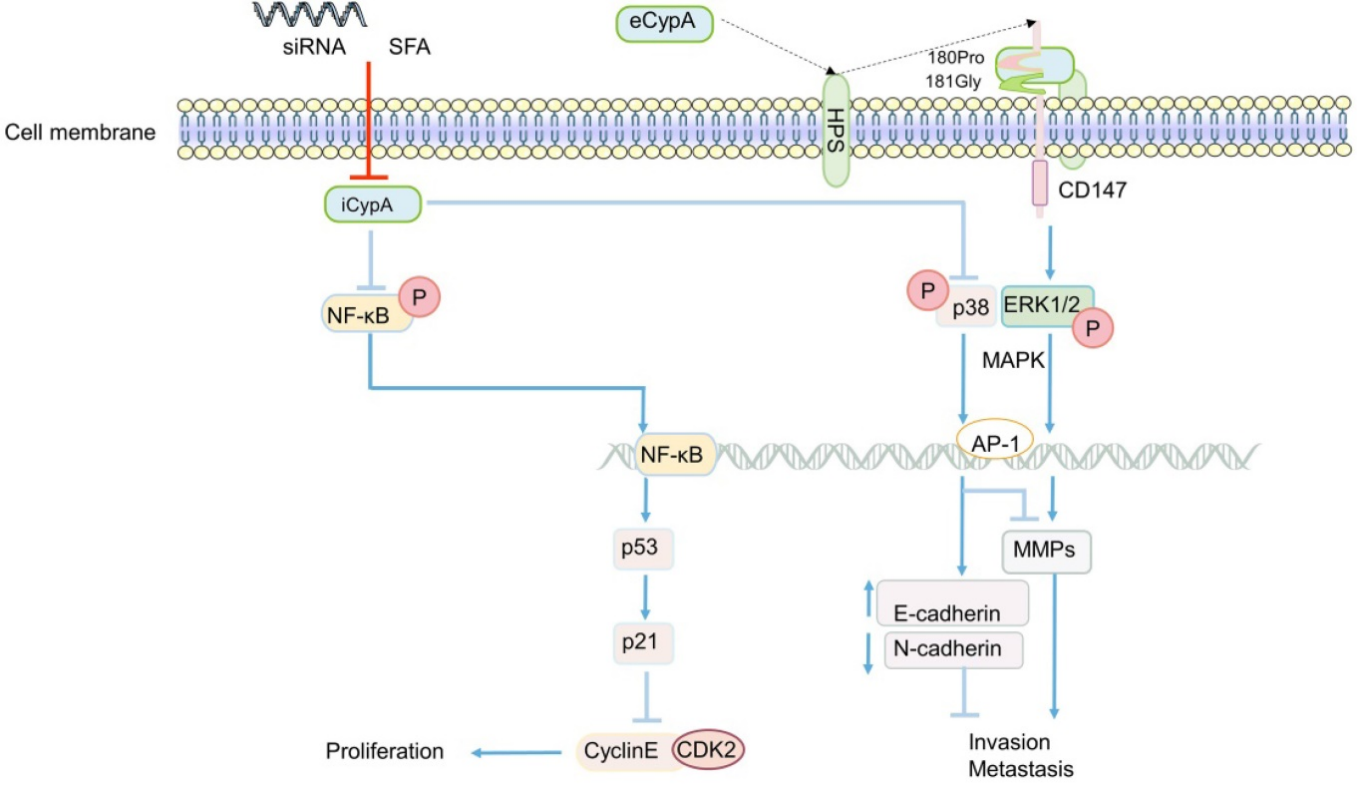
** The regulatory role of CypA in CRC.** eCypA and iCypA have different effects on NF-κB and MAPK pathways. Both iCypA and eCypA can respectively regulate CyclinE-CDK2, EMT and MMPs through these two signaling pathways, which have different effects on CRC.

The role of CypA in CRC generally focuses on the proliferation, invasion and metastasis of cancer cells. The effect of eCypA on CRC is also closely related to the signal regulation of CD147 receptor. The combination of eCypA and CD147 on cell membrane activated downstream pathways through ERK1/2 122, 158, and the activation of MAPK promoted tumor metastasis 34 (Figure 3). It is speculate that eCypA may play a role in the proliferation and metastasis of CRC by binding to CD147 and regulating the downstream MAPK signaling pathway. Meanwhile, a number of experiments have shown that the regulation of MMPs in tumors was controlled by the activation of the p38MAPK signal 159-162, and played an important role in promoting the invasion and metastasis of cancer cells 35, 159-162. Consistently, recent studies found that the activity of MMP-9 promoter was markedly enhanced in CRC 163 (Figure 3). In summary, it can be speculated that the combination of eCypA and CD147 may also regulate the expression of MMPs through the MAPK signaling pathway, which has a certain effect on the invasion and metastasis of CRC or other cancer cells (Figure 3).

Apart from eCypA, the inhibition or knockout of iCypA also affected the proliferation, migration and invasion of CRC. Early studies discovered that sanglifehrin A (SFA, one kind of immunosuppressor) inhibited the proliferation of macrophages through iCypA 124. Similarly, SFA was also found to inhibit the proliferation of human colon cancer cell HCT-116 after binding to iCypA 164. However, unlike the inhibition of ERK1/2 activity in macrophages (Figure 1) 124, SFA activated NF-κB signal after inhibiting iCypA in colon cancer cells. Subsequently, NF-κB promoted the transcription of tumor suppressor gene p53 in cancer cells, stimulated the increase of p21 expression, and inhibited the activity of cyclinE-CDK2, thereby inhibiting the proliferation of colon cancer cells 164 (Figure 3). Based on the results mentioned above, it is suggest that the inhibition of iCypA not only negatively regulates the migration and proliferation of immune cells 124, but also relates to the inhibition of CRC cell proliferation 164. However, the difference may be that iCypA has different effects on CRC cells and immune cells such as macrophages (Figure 1 and 3).

Nevertheless, in a recent study to explore the effect of CypA on the progression of colon cancer 165, the invasion and metastasis of SW480 cells were significantly inhibited after the iCypA gene was knocked out, but there was no effect on cell proliferation (Figure 3). Simultaneously, there was up-regulation of E-cadherin (regulating the epithelial properties of cells) and down-regulation of N-cadherin (regulating the mesenchymal properties of cells) 165, indicating that iCypA may promote the invasion and metastasis of colon cancer cells by regulating epithelial mesenchymal transition (EMT). It is worth mentioning that, similar to the phosphorylation of NF-κB signal induced by the inhibition of iCypA 164, the knockout of iCypA also caused the activation of p38MAPK (Figure 3). And the use of p38 inhibitors increased the invasion number of iCypA knockout colon cancer cells 165, which once again proves the previous conjecture that the inhibition or knockout of iCypA in CRC cells may promote the activation of NF-κB and MAPK signals. But, it may have the opposite effect in immune cells. Interestingly, the expression and release of MMPs involved in cell invasion were also examined. However, these MMPs did not change after iCypA knockout, even lowered than the detection limit 165 (Figure 3). This seems to be in contradiction with many experimental results indicating that the activation of MAPK promotes the expression of MMPs 34, 121, 134-136, but this suggests that eCypA and iCypA may have different effects on MAPK in different cancer cells (Figure 3). Additionally, the expression of MMPs cannot exclude the possibility of other signal pathway regulation, which needs further study. But it is certain that CypA does play an important role in the invasion and metastasis of CRC.

### CypB

In cancer biology, CypB is associated with the malignant progression and regulation of a variety of tumors 166-171, but its research in CRC is rarely reported.

**Figure 4:**
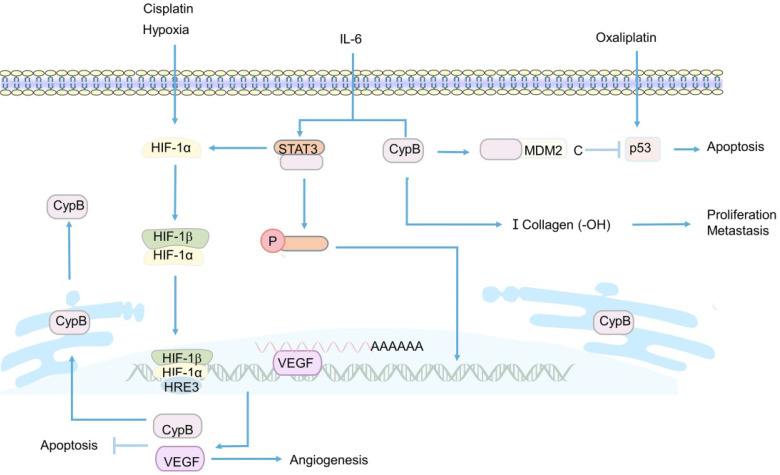
** Related mechanisms of CypB involved in CRC.** HIF-1α promotes the transcription and expression of VEGF and CypB, thereby inhibiting hypoxia and cisplatin-induced apoptosis in CRC. Overexpression of CypB can bind to STAT3 and induce its phosphorylation and nuclear transfer in the presence of IL-6, and also positively promotes the transcription of HIF-1α and the formation of type I collagen. p53 upregulates the expression of CypB, the interaction between CypB and N-terminal of MDM2 can degrade p53 protein and inhibit oxaliplatin induced apoptosis of CRCr cells.

In the last decade, Sung Soo Kim et al. have been devoted to the study of CypB 108, 152, 172. In 2011, they first studied the induction of CypB under hypoxia and its function in tumor cells *in vivo* and *in vitro* 152. Their sesults showed that CypB regulated angiogenesis though hypoxia inducible factor-1α (HIF-1α)-mediated vascular endothelial growth factor (VEGF), and protected tumor cells including liver cancer and colon cancer cells from stress-induced apoptosis, including hypoxia and cisplatin-induced stress 152 (Figure 4). These results suggest that CypB may be a new candidate target for the development of anti- hepatocarcinoma and colon cancer, which also lays a certain foundation for subsequent researches of CypB in chemotherapy resistance of CRC patients. However, in this study, the relevant mechanism of hypoxia-induced up-regulation of CypB expression was not deeply explored. In 2015, they demonstrated for the first time that hypoxia up-regulated the transcription of CypB by activating transcription factor 6 (ATF-6) 172, thus elucidating the mechanism relatively completely. In recent years, they began to study the mechanism of CypB regulating chemoresistance in CRC 108. The results showed that tumor suppressor gene p53 wild-type (p53WT) up-regulated the mRNA and protein levels of CypB, and overexpressed CypB interacted with ubiquitin E3 ligase (MDM2), so as to make p53WT ubiquitination degradation and short its half-life, thus inhibiting oxaliplatin-induced apoptosis of CRC cells (Figure 4). On the contrary, CypB knockout prolonged the half-life of p53WT and stimulated apoptosis of cancer cells 108. These results suggest that CypB is an effective target for improving chemoresistance in patients with CRC. In conclusion, these experiments suggest that CypB is regulated by oncogene transcription factors such as HIF-1α, ATF-6 and p53WT, which may be related to ER stress and extensive signaling pathways, and determine the role of CypB in chemotherapy resistance of CRC.

Apart from participating in the regulation of chemoresistance in CRC, CypB was found to play a critical role in the invasion and metastasis of CRC 153, 156. CypB was knocked out by RNA interference plasmid in CRC cell, the results showed that the migration and invasion abilities of cancer cells were significantly reduced 156 (Figure 4). However, the specific mechanism of CypB promoting the migration and invasion of cancer cells was not revealed in this study. A recent study showed that CypB silencing reduced the proliferation, invasion and migration of colon cancer *in vivo* and *in vitro* by blocking IL-6-induced signal transducer and activator of transcription-3 (STAT3) phosphorylation and nuclear translocation 153 (Figure 4). Besides, CypB silencing also blocked the hydroxylation of type I collagen and the formation of strip bands, thereby inhibiting the metastasis of cancer cells 153 (Figure 4). In conclusion, STAT3/CypB/collagen regulatory axis may play a crucial role in the development of CRC, and CypB may be an effective target for preventing the proliferation, invasion and migration of CRC.

### CypD

CypD is a component of mPTP. The role of CypD in mPTP has been widely used in experiments in recent years. It seems that the effect of CypD on CRC also revolves around cell death induced by mPTP 173-176. Some chemotherapeutic drugs have been found to promote the combination of mitochondrial ANT-1 and CypD, reduce the mitochondrial membrane potential, promote the opening of mPTP, and exert a toxic effect on CRC cells through mitochondrial programmed necrosis, inducing necrosis of cancer cells, but not apoptosis (Figure 2). Consistently, the toxicity was significantly attenuated when CypD inhibitors or corresponding siRNA were used 173, 174. In mouse, after CsA treatment, the inhibitory effect of chemotherapeutic drugs on the growth of HCT-116 xenograft was significantly weakened 174. Correspondingly, the overexpression of CypD significantly enhanced the sensitivity and cytotoxicity of CRC cells to chemotherapeutic drugs 174. This indicates that CypD may play an important role in the cytotoxic effects of chemotherapeutic drugs on CRC cells, and this effect is closely related to mitochondrial programmed necrosis. In the study of Chunxian Zhou et al., it was also found that the opening of mPTP stimulated by icariin (ICT, a chemotherapeutic drug), was induced by JNK activation pathway 173, suggesting that JNK signaling pathway may be one of the regulatory mechanisms of CypD involved in chemotherapy-induced CRC cell necrosis (Figure 2).

However, it is interesting that in addition to mitochondrial programmed necrosis, a new anti-cancer drug candidate has been found to induce apoptosis of CRC cells by disrupting mPTP 175. Erastin was a VDAC-1 binding small molecule that promoted VDAC-1 binding to CypD, cytochrome C release and regulated mPTP opening, thereby inducing mitochondrial oxidative stress and caspase-9-dependent apoptosis to produce cytotoxic effects on various CRC cells (Figure 2). CypD inhibitors significantly attenuated the drug-induced cytotoxicity and apoptosis 175. This not only indicates the emergence of a potential new drug against CRC, but also suggests that CypD may also be involved in the drug-induced apoptosis of CRC cells by regulating mPTP. To sum up, CypD can participate in the cytotoxic effect of drugs on CRC not only through the necrosis, but also through the apoptosis. The difference of these pathways may be related to the types of drugs, the molecules bound to CypD, and the ways to induce the opening of mPTP.

In a recent study, it was found that Ganoderma acid D (GAD) induced the deacetylation of CypD by up-regulating the level of mitochondrial deacetylase Sirtuin 3 (SIRT3) in a dose-dependent manner, which changed the open state of mPTP, thereby inhibiting the Warburg effect of colon cancer cells and causing cells death 176. This suggests a possible new way and mechanism for CypD to participate in drugs to inhibit CRC, but the inhibitory effects of different drugs on CRC cells are always related to the involvement of CypD in the regulation of the open state of mPTP.

## Summary

In general, Cyps is highly expressed in IBD and CRC, and even has a certain correlation with the course of disease and prognosis, which suggests that Cyps may be a diagnostic and prognostic indicator. For IBD, Cyps can play a role in inflammation through the secretion of inflammatory factors, apoptosis, autophagy and necrosis of immune cells. In CRC, Cyps is mainly involved in invasion, metastasis, apoptosis, necrosis and drug resistance of cancer cells. The mechanisms of Cyps in these two diseases are generally focus on the signaling molecules such as MAPK, NF-κB and mitochondrial programmed death, and the specific mechanisms remain to be further explored. However, at least it is certain that Cyps will play an important role in the future research on IBD and CRC, and provide effective clues for disease treatment targets or new drug development.

## Abbreviations

CRC: colorectal cancer
IBD: inflammatory bowel disease
Cyps: Cyclophilins
UC: ulcerative colitis
PPIase: peptidyl prolyl isomerase
CsA: Cyclosporin A
NF-AT: nuclear factor of activated T cells
CypA: Cyclophilin A
CypB: Cyclophilin B
CypC: Cyclophilin C
CypD: Cyclophilin D
Cyp40: Cyclophilin 40
CypNK: Cyclophilin NK
iCypA: intracellular CypA
VSMC: vascular smooth muscle cells
EC: endothelial cells
eCypA: extracellular CypA
ER: endoplasmic reticulum
eCypB: extracellular CypB
HIV: human immunodeficiency virus
mPTP: mitochondrial permeability transition pore
VDAC: voltage-dependent anion channel
ANT-1: adenine nucleotide translocator-1
CD: Crohn's disease
CypJ: Cyclophilin J
DSS: Dextransulfatesodium
NF-κB: nuclear factor kappa B
LPS: lipopolysaccharide
CD147: differentiation cluster 147
MMPs: matrix metalloproteinases
TIMP-1: tissue inhibitor of matrix metalloproteinases-1
EMMPRIN: extracellular matrix metalloproteinase inducer
HPS: Heparan sulfates
GAG: Glycosaminoglycans
mPT: mitochondrial permeability transition
NSAID: non-steroidal anti-inflammatory drugs
PPIL3: PPIase-like 3
NZF: Npl4 zinc finger
TNM: tumor, node, metastases
SFA: Sanglifehrin A
EMT: epithelial mesenchymal transition
HIF-1α: hypoxia inducible factor-1α
VEGF: vascular endothelial growth factor
ATF-6: activation of transcription factor-6
p53WT: p53 wild-type
MDM2: ubiquitin E3 ligase
STAT-3: signal transducer and activator of transcription-3
ICT: Icariin
GAD: Ganoderma acid D
SIRT3: Sirtuin 3
